# Return to Status Quo Ante: The Need for Robust and Reversible Pandemic Emergency Measures

**DOI:** 10.1017/S096318012000078X

**Published:** 2020-10-02

**Authors:** STEPHEN RAINEY, ALBERTO GIUBILINI

**Keywords:** Corona virus, pandemic response, ethics, political legitimacy

## Abstract

This paper presents a normative analysis of restrictive measures in response to a pandemic emergency. It applies to the context presented by the Corona virus disease 2019 (COVID-19) global outbreak of 2019, as well as to future pandemics. First, a Millian-liberal argument justifies lockdown measures in order to protect liberty under pandemic conditions, consistent with commonly accepted principles of public health ethics. Second, a wider argument contextualizes specific issues that attend acting on the justified lockdown for western liberal democratic states, as modeled on discourse and accounted for by Jürgen Habermas. The authors argue that a range of norms are constructed in societies that, justifiably, need to be curtailed for the pandemic. The state has to take on the unusual role of sole guardian of norms under emergency pandemic conditions. Consistently with both the Millian-liberal justification and elements of Habermasian discourse ethics, they argue that that role can only be justified where it includes strategy for how to return political decisionmaking to the *status quo ante*. This is because emergency conditions are only justified as a means to protecting prepandemic norms. To this end, the authors propose that an emergency power committee is necessary to guarantee that state action during pandemic is aimed at re-establishing the conditions of legitimacy of government action that ecological factors (a virus) have temporarily curtailed.

At the beginning of the Corona virus disease 2019 (COVID-19) outbreak in Wuhan, China, measures were reportedly first put in place to limit information about the virus being spread. This led to the contagion going undetected for a few weeks, or even months. Once it became clear that the contagion was out of control and becoming a public health emergency, local Governments had to enforce a total lockdown of major population centres, with the aim of stalling virus spread. But it was too late. The infection had in the meantime spread globally. Pretty soon, other countries would emulate this lockdown approach, to greater or lesser degrees, and more or less quickly, in response to the virus. In many countries, people were prevented from leaving their home if not for essential activities (buying food, essential work, and sometimes some form of exercise). The production and economic systems of many countries were paused for months.

**Figure 1.**
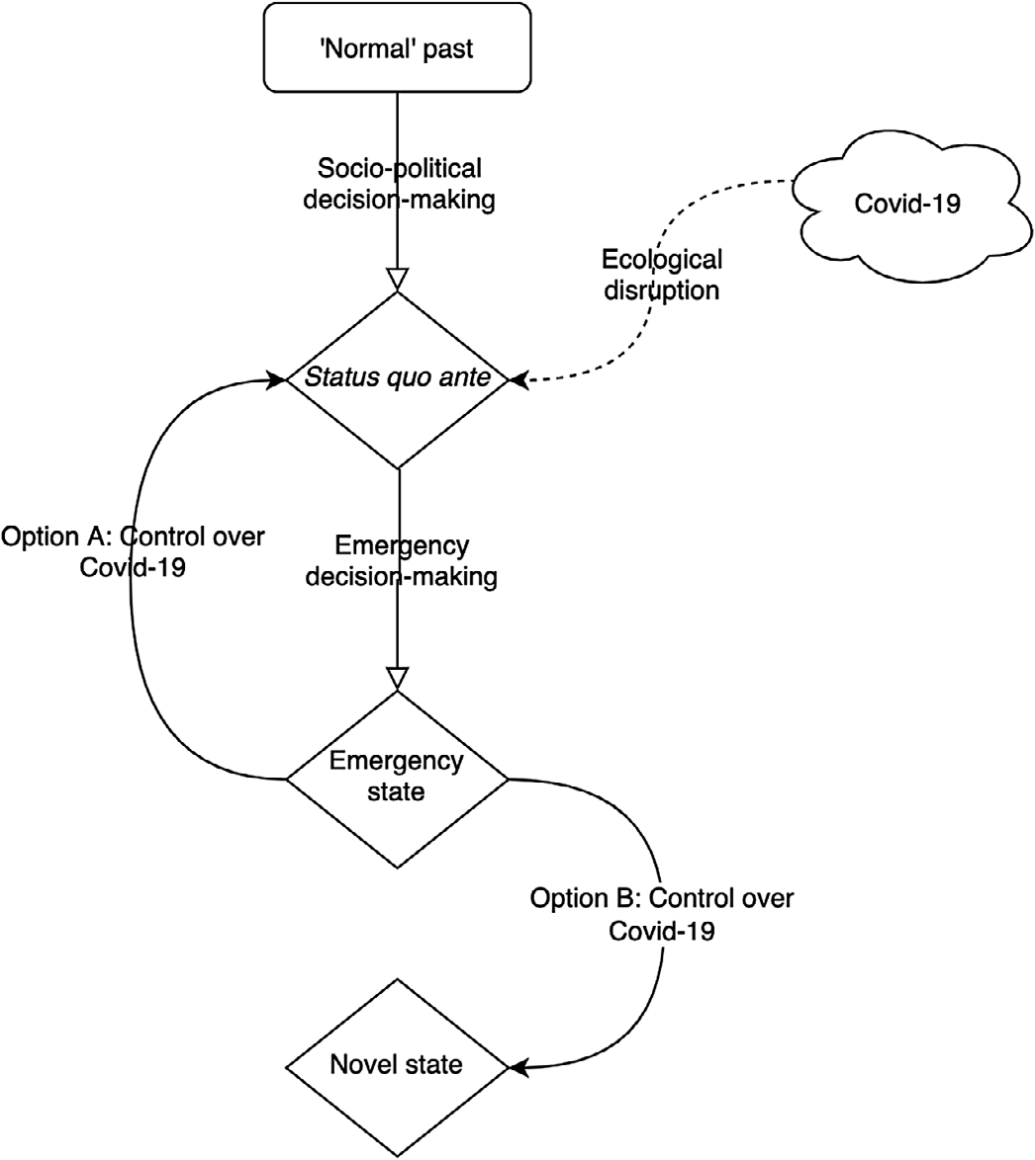
Simplified picture of pandemic response: From a “normal” state, standard decisionmaking produces a *status quo*, in which features all the established sociopolitical norms and procedures expected by a citizenry. Once disrupted, an emergency state is justified, but which requires the suspension of norms and procedures. Exit from the emergency state produces at least two options. One is a return and another is a novel state. Only that *status quo ante* appears as the result of expected norms and procedures, and so only a return is legitimate. If a novel state is desirable, it must result from the *status quo ante* for this reason. The emergency state is justified only to the extent that it is a response to a disruption. It is not a basis for transition to a novel state, although learning that takes place in it might inform subsequent activity in the status quo ante.

At the time of writing, many countries all over the world have started easing the lockdown measures: but there are reports of new outbreaks in areas where the virus seemed to have disappeared; and experts warn that we are very far from having natural herd immunity.^1^ ^,^^2^ If that happens, important decisions will have to be made about whether and how to revert to some form of lockdown, and what principles such decisions should be based on. The path back to normality is likely to present some bumps and not be a linear one, and it is important to identify what principles should guide decisions about relaxing and restricting measures.

The Oxford COVID-19 Government Response Tracker (OxCGRT) set out with the aim of cataloguing the variety of responses as they occur, and evolve. Using 11 markers, OxCGRT have developed a “stringency” metric that can indicate from 0 to 100 the strength of governmental response to the first wave of the pandemic.^3^ Stringency draws upon requirements, such as school or workplace closures, movement restrictions, and fiscal measures, including whether such requirements are suggested or mandated by law. The picture of stringency varies, but trends can be seen particularly where COVID-19 cases rise. The stringency of measures trends upward as cases multiply. What is common across the majority of responses to the virus is the suspension of certain social norms, freedoms, and procedures in the service of dealing with the emergency.

The trend suggests that more stringent measures are perceived as more justified, at least by policymakers, as the severity of the pandemic becomes clearer. We suggest stringency is justified from the outset, but not without qualification. When implementing very restrictive measures that entail temporary infringements on certain rights, it is important that Governments and authorities in general, at least in democratic societies, have the strongest ethical justification possible. Although this should always be a requirement for policy decisions, it becomes more stringent, the more policy decisions infringe upon those fundamental rights that constitute the backbone of liberal democratic societies, and in particular, certain fundamental freedoms. We will argue that appealing to the emergency situation and to the death toll of the pandemic, although necessary, is not sufficient to provide the best ethical justification possible. What we need in addition, is an appeal to the kinds of values that we want to protect by preventing even more deaths and by alleviating the burdens on healthcare systems.

Once we consider these values, we see that restrictive measures cannot be fully justified without a plan to restore those values once the emergency is over. In other words, there can be no lockdown strategy without an adequate postlockdown view, if we want Governments’ actions to be ethical. Clearly, what the world will look like after the lockdown, or after any future lockdown following a second wave, and what precise measures will be more appropriate is difficult to predict at the moment. Much will depend on epidemiological considerations about the virus’ spread, on social and psychological considerations about how people will respond, and on economic considerations about how different countries will be able to cope with the production, economic, and financial crisis. However, as much as possible, countries should ensure that citizens who have some of their fundamental rights infringed upon for the sake of the collective good, and especially for the good of certain population groups (e.g., the elderly and others more vulnerable to COVID-19) have as clear an idea as possible of what steps will be taken to ensure that all of their rights will be restored as soon as possible.

We will argue for these claims first on the basis of a liberal argument drawing upon John Stuart Mill’s classic account of liberty. This will set out justifications for restrictions on freedoms in the name of preventing predictable harms. Although this is compelling in its own right, we will go on to argue further that it ought to be seen as nested within a broader account of sociopolitical norms, well accounted for by Jürgen Habermas. In this broader account, sociopolitical norms are located within established governance arrangements. Aside from an abstract argument for the suspension of norms in the name of preventing harm, an argument must be presented that can justify such a suspension from this more situated perspective. This is especially important when it comes to considering what happens when the emergency situation passes, and a return to something like normal is considered.

## Ethical Justifications

One of the core principles of liberal democracies is the widely used and abused slogan borrowed from John Stuart Mill’s *On Liberty*: “The only purpose for which power can be rightfully exercised over any member of a civilized community, against his will, is to prevent harm to others.”^4^

The principle is not as straightforward as it might at first glance seem. Most notably, there can be different views about which liberties of an individual may permissibly be constrained for the sake of which liberties and rights of other individuals. And there are different implications of the principle when it comes to collective harm, that is, harm that results from some form of collective behavior, where each individual contribution does not make a (significant) difference. In general, though, there is a hierarchy that is widely accepted at least when it comes to regulating individual behavior that can result in direct harm: right to life is almost invariably at the top of this hierarchy, and right to a decent minimum of healthcare is normally just behind (there are noteworthy exception, of course: in the United States, the right to healthcare does not seem to rank very high, and in theocratic countries, the right to life is subordinate to religious dogmas). Freedom of movement and association, right to privacy, and other basic rights are of course taken to be very important, but not *as* important. The justification for this hierarchy is quite straightforward: life and a decent minimum health are preconditions for enjoying the benefits of the other (human) rights. For instance, you need to be alive and in relatively good health to enjoy the benefits of freedom of movement. This ranking implies that, when life or provision of basic healthcare are at risk, it seems reasonable to temporarily sacrifice—to the lowest degree possible—things like freedom of movement and privacy.^5^ ^,^^6^ ^,^^7^

Of course, this is only a general point that is not—and perhaps should not be—applied consistently. For instance, a great deal depends on the directedness of the causal connection between human behavior and its consequences. The intuition that grounds most of the legal frameworks in liberal democracies is that infringements on freedom of movement can be justified when people’s movements more directly put other people’s lives at risk, for example, when someone risks infecting someone else with a potentially lethal disease. Infringements are less justified when the link is more indirect, for example, when people contribute to polluting the environment through constant travelling, thus increasing the risk of general respiratory disease, and cancer for everyone.

Simply put, by the very same hierarchy of values that informs liberal democracies and considering the direct impact that people’s freedom of movement can have on the risk of life for others, an ethical response to the COVID-19 pandemic, and future such emergencies, is to shut down as much of normal life as possible and as necessary to reduce the spread of the contagion in a way that minimizes the number of deaths and the load on healthcare systems. It is the optimal way to protect health as well as the possibilities of returning to normal life sooner, by sacrificing values that rank lower in the widely shared and intuitive hierarchy that underpins most social and legal norms. Were infections to soar despite a shutdown of normality, it would probably have been worse without, as the aforementioned estimate suggests. If things were to go well, and infection rates stall, the shutdown might at some point look needless to some. But its apparent pointlessness would have been caused by its own efficacy—a destiny that lockdown shares with other public health interventions, such as effective vaccination policies. And if it really was needless, there would be no way to know. Although it is true that some countries, such as Sweden, have implemented much milder measures than others, such as the United Kingdom or Italy, we cannot really compare different measures implemented in different countries to assess the effectiveness of lockdowns. Local circumstances make comparisons not particularly useful or even meaningful. For instance, if a country like Sweden has a better healthcare system than other countries where strict lockdowns have been implemented, then it can afford a higher rate of infections without risking a collapse of the system.

A widely accepted principle in regulating public health interventions is the so-called “principle of least restrictive alternative” (PLRA).^8^ Simply put, the principle demands that we implement the policy that entails “the least intrusion on personal rights and freedoms” among those that can achieve the relevant public health goal.^9^ In this case, the goal is to contain the contagion in a way that would allow healthcare systems operating in regimes of scarce resources to save as many lives as possible, or, where triage is in place, as many expected life years possible or whatever other units are used as a prioritization criterion. The principle seems rather intuitive. The Nuffield Council on Bioethics has formulated an “intervention ladder” that ranks a series of possible types of public health interventions according to the PLRA, from the least restrictive (doing nothing or simply providing information) to the most restrictive ones.^10^ Restrictions of freedom of movement through Government action are, needless to say, at the bottom of the ladder, as a measure of last resort. And yet, emergency situations are “emergency” precisely because they might require measures of last resort. Informing people about the importance of staying home, inviting or nudging people to stay home, are simply not effective in most contexts. For instance, the United Kingdom had to scale up the level of state coercion after simply asking people to avoid certain types of social interactions and behave responsibly did not work. Hence, the widely accepted hierarchy of values justifies, in a case like the COVID-19 pandemic, going down the ladder and enforcing the kind of lockdowns we are seeing in many countries. This is consistent with the PLRA precisely because of the emergency situation and the value we attribute to what is at stake: The lives of many people and the capacity of states to provide basic healthcare.

Another way to frame this ethical justification for very restrictive emergency measures is to appeal to utilitarianism. Whether or not utilitarianism is the best ethical theory to regulate decisionmaking in normal everyday life—something most people, rightly or wrongly, would object to—some have argued that a principle of maximization of expected utility is the most apt to regulate policy in disaster situations,^11^ for example in triage decisions.^12^ This makes sense if we think that disaster situations are disruptive of the normal circumstances that favor the balancing of a plurality of values (e.g., expected utility, fairness, liberty). Such balancing seems a better way of handling ethical and political decisions in normally functioning democratic settings, at least if we assume that pluralism is a preferable approach to moral disagreement in liberal democratic societies. However, disaster situations, including the COVID-19 pandemic, are by definition not normal circumstances, and many of the basic arrangements of liberal democracies (for instance, free movement) are curtailed anyway.

A utilitarian approach in emergency situations can be justified by appealing to the distinction between ideal and nonideal theories. The distinction is widely discussed in the Rawlsian tradition of political philosophy,^13^ where what makes a theory “ideal” is the (unrealistic) assumption of widespread compliance.^14^ The distinction has been given alternative interpretations in political philosophy, almost invariably with reference to Rawls’ approach to a theory of justice.^15^ Here, we understand the distinction more specifically in the way it is has sometimes been applied in bioethical discussion of disaster situations. Within this perspective, “[i]deal theory typically abstracts from the specifics of a situation or issue to identify general principles that allow the right answer to be determined.”^16^ In this view, utilitarianism is an ideal theory because it tells us that, no matter the circumstances, the maximization of expected utility is the right answer to whatever ethical question we may raise. Now, the common view is that ideal theories are unsuitable in situations of tragedy or disaster, where pursuit of certain goods inevitably requires sacrificing others. Rather, many believe that nonideal theories are required where “not all wrongs can be rectified, not all losses can be compensated, not everything can be repaired or replaced, and—especially given the limits of psychological resilience—not everyone can recover.”^17^

We take issue with this view and we claim the opposite is true. Ideal theory that has one general principle about the right and the wrong is precisely what we need in situations, in which balancing different principles and values seems unfeasible. We have a *prima facie* right to free movement and a *prima facie* right to health and to life. We normally balance these principles against each other, but in a situation like the COVID-19 pandemic, this is not possible: free movement would seriously threaten many people’s health and life. So, we need one criterion to rank these values and determine which one should take priority. Utilitarianism offers such a criterion: we need to maximize expected utility, that is, saving as many lives as possible and as many healthcare resources as possible, until doing so entails too large a burden for people’s wellbeing (e.g., in terms of mental health of people in lockdown). Utility is maximized not only because lives are saved and healthcare delivery is preserved, but also because saving lives and preserving healthcare delivery will create the circumstances where people will be able to enjoy their freedoms in the future: life and health are precondition for whatever one thinks represents the measure of “utility” (pleasure, happiness, preference satisfaction, some objective goods including certain rights, etc.).

One of the reasons why many find utilitarianism controversial is that the principle of maximization of expected utility in normal circumstances conflicts with many other values and principles that we rightly hold dear, for example, with certain freedoms. Balancing these different principles against each other is reasonable in normal times: we want overall wellbeing, but we also want certain freedoms respected. However, in a situation where respecting these other values and principles would come at too high a utility cost—in terms of people’s health and lives—the balancing becomes unreasonable and the application of an ideal theory like utilitarianism becomes way more plausible. We always want to maximize expected utility. It is just that normally we want to do that, *compatibly with respecting other values*. That is, expected utility is just a value among others. In an emergency like the COVID-19 pandemic, respecting these other values would entail too large a cost.

As said above, emergencies make it the case that in order to preserve things that we value more, such as life and provision of basic healthcare, we need to sacrifice certain individual rights and liberties whose value is ranked lower. Thus, emergency removes one of the common objections to utilitarianism, namely that it would demand sacrificing individual rights if this is necessary to maximize expected utility. Since we place more value in saving lives and guaranteeing healthcare provision, and since there is no alternative to sacrificing certain individual rights to achieve those targets, maximizing expected utility (as determined by what we value the most) by sacrificing certain individual rights is less problematic than it would be in normal circumstances.

Swift and extensive shutdown presents a clear, simple, although severe message for the public. This can help alleviate the possibilities for mixed-messaging, especially those that might result from the emergence of differing approaches, for example, across European nations.^18^ Swift and maximal shutdown also forestalls any ideological prevarication, wherein rhetoric hinders action—the state need not begin to reflect on the nature of political freedom ahead of taking action. Rather, action is taken that is proportionate to threat, and justified in light of that threat, at least according to the threefold argument we have provided, that is an argument based on (1) the commonly accepted ranking of values, (2) the principle of least restrictive alternative, and (3) the legitimate appeal to utilitarianism in emergency situations like this.

In one sense, anything done swiftly in the face of pandemic might look like over-reaction from this side of the emergency. But thinking about life afterwards, most responses will probably look complacent. What many Governments have fortunately come to realize, and what some of them have unfortunately come to realize too late, is that suspension of established and desirable norms is not only ethically justified but indeed ethically required once the emergency nature of the situation is acknowledged.

This discussion has focussed on the justification of restricting rights in the light of emergency situations. However, the pausing and restarting of rights is quite an abstract manner of considering what is practically at stake here. The Millian-liberal argument so far has to be contextualized, where complex modern democracies are at stake, to recognize that “liberty” in such contexts is mediated by a web of legal/social norms arrived at via democratic discourse. Besides the moral argument, given states that are not *ethocracies* but *democracies*, further argument is required concerning how ethical suspension of norms ought to proceed. It is not abstract liberty at stake, but sociopolitically situated freedom. This situatedness is best recognized in emergency conditions by signaling those conditions with specific, limited, robust, and reversible measures.

What follows is a broader discussion of how sociopolitical norms also figure in western, liberal democracies. Having considered this, we will argue that the justificatory arguments just provided require tailoring to the specifics of democratic states as based on a model of discursive legitimacy. As the previous discussion of the utilitarian justification for the lockdown already foreshadowed, this includes the necessity of a way back to the decisionmaking procedures and the norms they generated in the *status quo ante*.

## Normative Complexity

Contemporary western democratic societies are not composed of homogenous groups of citizens who can be expected to coordinate their activities without some kind of procedure. There are a variety of sources for norms in such societies. Jürgen Habermas’ way of accounting for the procedure is to model social coordination upon various dimensions of linguistic communication.^19^ Language is a medium whereby agreements are made, disagreements understood, and problems laid out for consideration. Accounting for the ways in which citizens may coordinate their activities amid social diversity is possible through anchoring an account of the state in a model of discussion.

A sociopolitical order with a discursive format displaces power struggle and conflict into a mode that can produce agreements about what is best, given the available arguments.^20^ This is a way of characterizing the broad nature of western, liberal democracies as we know them. The realization of the discourse varies, as does the quality of its form. Nevertheless, ideally, citizens are presented with information on political decisions to be made, evidence from reliable sources, commentary from a free press. Through dialogue, they can go on to adopt a stance toward matters of the day. Elections typically mark the culmination of these kinds of deliberations, being the point when decisions are made about how to go on. Overall, the entire endeavor is one of discourse. The force of argument replaces the notion of obeisance to a least-worst protector in a state of nature, as a Hobbesian state might prescribe. From the discursive state legitimacy can grow, as reasoned positions can be adopted, rather than being driven solely by fear and survivalism.

The state provides basic, legal norms that ultimately constrain liberty to an extent that is deemed necessary to protect other people’s fundamental liberties and other rights (in line with Mill’s harm principle from above). But aside from the basic laws generated and enforced by the state, a plurality of norms emerges in context-specific domains. For instance, economics, science, industry, and so on, each has a structure that generates its own norms that serve to set a course for that sector’s development. As each is interlinked with the rest, moreover, these norms are also interlinked and grow in interdependence. This presents a complex picture, but the main takeaway is that the state is by no means the sole source of norms in western, liberal democracies.^21^

This way of analyzing liberal democracies is neatly used in Habermas as he distinguishes the lifeworld and system. This is a distinction that identifies different dimensions of social interaction and cooperation. The lifeworld is the broad context in which social actors act: it is the world as it is known by them, in which they understand their possibilities from a social point of view.^22^ Importantly, the lifeworld is steered by discursive agreements. The lifeworld is itself reliant upon the functioning of various system mechanisms, like economics, and political administration.^23^ These are not primarily steered by wide-ranging public discursive agreement, but by their own norms. Without the good functioning of the enabling subsystems, the lifeworld would lack practical realization.

The lifeworld is comprised of shared cultural resources and institutional arrangements that can coordinate patterns of action, as well as person-specific value systems such as morality. This shared background enables actors to cooperate on the basis of mutual understanding. Alongside the lifeworld has evolved system mechanisms. System mechanisms enable the lifeworld to function by providing coordination functions not rooted in shared assumptions or cultures, institutions, or personal value systems. Because large and complex western democracies cannot expect to manifest coordinated societies on the basis of such shared resources, specific system mechanisms of coordination emerge, which mediate coordination functions *via* money and power.^24^

Without a well-regulated economy, for example, some familiar practices of “steering” social development through capital investment would cease to be effective. This could entail the reappraisal of what may be taken for granted in everyday life about a society’s self-conception and idea of progress. It would also lead to a deficit in administrative power as political decisions about priorities would lose an efficient means of being put into operation. The state itself requires administrative power, and the ability to prioritize in order that it can constrain any social actor, as when criminality is punished with imprisonment, for instance. Money and power can be seen as system mechanisms underwriting a lifeworld more generally coordinated through discursive agreement. Nevertheless, the lifeworld ought ordinarily to have a general position of authority over the subsystems. Discursive agreement is that which can legitimately steer a state. Ordinarily, this should mean that the discourse that ultimately shapes society has an ultimate means of steering subsystems to remain as providing opportunities in the lifeworld, not the other way around.^25^

The administrative arrangements arrived at represent collective efforts (hopefully best efforts) to establish a cooperative mode of coexisting among the varieties of forces that might represent resistance to those efforts. The incursion of ecology into that lifeworld, by means of a virus, might seem surprising, but history has seen earthquakes, tsunami, and disease before. Arresting the worst effects of social emergencies brought on by natural forces is necessary for the survival and wellbeing of each member of society. But returning to something like normal political decisionmaking after the emergency is just as necessary. This is because the political processes interrupted by natural emergency are the only ones from which legitimate political pronouncements can issue (as illustrated in Fig. 1). This provides a basis for supposing that emergency response should be delivered differently from the normal mode.

The legitimacy of pandemic response is based in the need to protect rights and freedoms gained by legitimate political means—discourse prior to the emergency—but these are *de facto* curtailed until the emergency is passed. This gives the emergency response a dual nature that must be scrutinized: on the one hand, it ought to be permitted to do whatever practicality demands to end the emergency. On the other, its constraints in clearing the ground for the reinstatement of the *status quo ante* cannot be forgotten. It can act counter to received norms as forced to by the virus, by becoming uncharacteristically utilitarian as suggested above for example, but cannot legitimately uproot a possibility for a return to prior norms. To do so would represent a contradiction, given its *raison d’être* is to counter the virus, on the basis that the virus would shatter the normality whose return they are convened to preserve.

## Delivering Emergency Response: The Need for an Emergency Power Committee

The state entity responding to pandemic through very coercive and restrictive measures ought to be recognized as having the right to govern in exceptional circumstances like this. The suspension of normal state functioning is necessary, given the emergency constituted by the virus. This power, however, ought to be clearly distinguished from the right to rule the nation. The emergency arrives not by way of any politically legitimating discursive channel, but by a matter of ecology. The response therefore cannot claim political legitimacy for the measures taken, merely the right to govern the effects created by deadly happenstance. The response is not delivered by the kind of state entity familiar in the lifeworld. It is more like a temporary shifting of lifeworld governance into a system mode in order to retain the possibilities of that lifeworld, despite extraordinary circumstances. It is in this context that the Millian, utilitarian approach described above becomes justified. The system-like response is centered upon preserving the possibility of lifeworld, and its inception arises from discursive activity in the lifeworld.

An emergency power committee, suitably scrutinized by existing departments of state, such as the UK’s Home Office, Office of National Statistics, the judiciary, and so on, separated from the established executive, would seem best placed to administer an emergency situation. This is because the committee itself could be inaugurated by standard political means as soon as a pandemic or similar emergency is recognized, and so derive legitimacy thereby. For the duration of its operation, it could draw upon this legitimacy to enact the measures necessary to combat the emergency, including curtailing the implementation of established rights and freedoms. This would be justifiable because the encroachment of the virus, a nondiscursive intervention, deforms the discursive nature of society by its very presence. The response may permissibly be constituted nondiscursively as it does not have a discursive means to confront a virus. Moreover, the measures it may take are taken in the service of protecting the continued possibility of the rights and freedoms familiar from the *status quo ante*. Given this, careful monitoring of the committee is required.

Public accounting for emergency measures is extremely important. In ordinary circumstances, the documenting of political discourse is one function of a free press, with another being the representation of public opinion. The press has a critical role in times of emergency in clearly staking out emergency response in light of established norms. These norms may be diminished to various degrees in terms of implementation for the duration of the emergency, but as the only valid norms for a given society, their power cannot be completely forgotten. These norms must inform ongoing appraisal of the emergency response in order to permit a return to the *status quo ante* once the emergency has passed. Likewise, the pre-emergency functioning of societal groups as represented by civil society organizations, charities, and other institutions has a role to play in holding that *status quo ante* in trust in order to inform a return. The nearest approach to this seen in the current emergency is that undertaken by New Zealand, which was swift to close borders and to set up response-monitoring bodies.^26^

These functions that would inform a return to normality are vital as there is no public duty to obey an emergency committee in postemergency circumstances. As suggested above, the *status quo ante* is the only perspective from which norms might justifiably be constructed (or curtailed). A change of norms in response to emergency is valid from that point of view, but only in terms of a deviation from normality—as a system-like mode of governance for the preservation of the lifeworld’s possibility. Because the deviation can be expected to be far-reaching, and full of risks of over-reach, there is special responsibility on those individuals, institutions, and other bodies representing pre-emergency lifeworld functioning to keep track of what happens. Close scrutiny permits subsequent audit, and thereby, a return.

A return to *status quo ante* is necessary even if certain changes in the emergency state are considered desirable also in a pre- or postemergency situation. Changes that are deemed desirable during normal times (e.g., universal basic income) need to be enacted via the pre-emergency administration, by the political means in place prior to the emergency. The emergency can *cause the desire* for change, but cannot justifiably *be the mechanism* of delivering the change. That would undermine the legitimacy of the change in using a fracture in discourse to begin another discourse. An interruption is not in itself a discursive contribution, it is a circumstance constraining such contributions. As such, it cannot itself ground a discursive move, so it cannot be a politically legitimate force.

## Conclusion

Norms have to be recast by an emergency authority in the light of pandemic. This is justified owing to the possibility of the virus undermining normal life. But given the unusual state of affairs, the uncomfortable measures that emerge, and the possibilities for overreach the critical roles of other elements of society at large require special emphasis. We need to ensure that a return to the *status quo ante* is an important part of the view informing emergency actions. As we have seen, there are strong ethical justifications for implementing restrictive measures as an emergency response. Such justifications are grounded in our widely shared hierarchy of values, in the principle of least restrictive alternative in public health, in utilitarianism, and in Habermas’ discourse ethics. However, both the utilitarian and, even more strongly, the Habermasian approach require that a view to a return to the *status quo ante* is encapsulated in the emergency response. To this end, we have argued that a special emergency power committee be created by normal democratic means to steer government response action toward that end.

## Notes

1. Wise J. Covid-19: Risk of second wave is very real, say researchers. BMJ 2020; 369.10.1136/bmj.m229432518177

2. Flaxman S, Mishra S, Gandy A, Unwin HJT, Mellan TA, Coupland H, et al. Imperial College COVID-19 response team. Estimating the effects of non-pharmaceutical interventions on COVID-19 in Europe. Nature 2020;584:257–61.3251257910.1038/s41586-020-2405-7

3. Blavatnik School of Government. *World’s First COVID-19 Government Response Tracker Launched Today*; 25 March 2020; available at https://www.bsg.ox.ac.uk/news/worlds-first-covid-19-government-response-tracker-launched-today (last accessed March 2020).

4.

Mill JS , et al. *On Liberty, Utilitarianism, and Other Essays* Oxford World’s Classics. New edition. Oxford: Oxford University Press; 2015, at 13.

5. Debevoise EW, Lindsay GN. *Siracusa Principles on the Limitation and Derogation Provisions in the International Covenant on Civil and Political Rights*; American Association for the International Commission of Jurists, Inc.; 1985.

6. European Commission. *Justice and Fundamental Rights, Text*; available at https://ec.europa.eu/info/policies/justice-and-fundamental-rights_en (last accessed June 2020).

7. Human Rights Watch. *Human Rights Dimensions of COVID-19 Response*; available at https://www.hrw.org/news/2020/03/19/human-rights-dimensions-covid-19-response (last accessed March 2020).

8.

Childress JF , et al. Public health ethics: Mapping the terrain. The Journal of Law, Medicine & Ethics 2002;30(2):173 10.1111/j.1748-720X.2002.tb00384.x.12066595

9.

Gostin LO , Wiley LF . Public Health Law: Power, Duty, Restraint. Berkeley, CA: University of California Press; 2016.

10.

Hepple B , Nuffield Council on Bioethics, eds. Public Health: Ethical Issues. London: Nuffield Council on Bioethics; 2007.

11.

Karadag OC , Hakan AK . Ethical dilemmas in disaster medicine. Iranian Red Crescent Medical Journal 2012;14(10):602–12. https://www.ncbi.nlm.nih.gov/pmc/articles/PMC3518976/ (last accessed 17 Sept 2021).23285411PMC3518976

12.

Wagner JM , Dahnke MD . Nursing ethics and disaster triage: Applying utilitarian ethical theory. Journal of Emergency Nursing 2015;41(4):300–6.2551020810.1016/j.jen.2014.11.001

13. Phillips M. Reflections on the transition from ideal to non-ideal theory. *Nous* 1985:551–70; John Simmons A . Ideal and nonideal theory. Philosophy & Public Affairs 2010;38(1):5–36.

14.

Rawls J. A Theory of Justice. Cambridge, MA: Harvard University Press; 2009.

15.

Valentini L . Ideal vs. non-ideal theory: A conceptual map. Philosophy Compass 2012;7(9):654–64. doi:10.1111/j.1747-9991.2012.00500.x.

16.

O’Mathúna D. Ideal and nonideal moral theory for disaster bioethics. Human Affairs 2016;26(1):8–17. doi:10.1515/humaff-2016-0002.

17.

Tessman L. Idealizing morality. Hypatia 2010;25(4):801.

18.

Anderson M , Mckee M , Mossialos E . Covid-19 exposes weaknesses in European response to outbreaks. BMJ 2020;368. doi:10.1136/bmj.m1075;32188590 Horton R. Offline: COVID-19—A reckoning. *The Lancet* 2020;395(10228):935. doi:10.1016/S0140-6736(20)30669-3.

19.

Habermas J . The Theory of Communicative Action: Vol. 1: Reason and the Rationalization of Society, T. McCarthy T, trans. Boston: Beacon (Original Work Published 1981); 1984.

20.

Habermas J . Between Facts and Norms: Contributions to a Discourse Theory of Law and Democracy, Studies in Contemporary German Social Thought. Cambridge, MA: MIT Press; 1996.

21.

Castells M . The Rise of the Network Society. Hoboken, NJ: John Wiley & Sons; 2011.

22. See note 17, Habermas 1984, at 335–37.

23.

Habermas J . The Philosophical Discourse of Modernity: Twelve Lectures. Hoboken, NJ: John Wiley & Sons; 2018.

24. See note 18, Habermas 1996, at 75.

25. see also Karl-Otto Apel. How to ground a universalisitc ethics of co-responsibility for the effects of collective actions and activities? Philosophica 1993;52(2):9–29.

26. Graham-McLay C, Roy EA. Tougher New Zealand rules on Covid-19 could set it apart as “normal” nation after lockdown. *The Guardian*; 31 March 2020, sec. World news; available at https://www.theguardian.com/world/2020/mar/31/tougher-new-zealand-rules-on-covid-19-could-set-it-apart-as-normal-nation-after-lockdown (last accessed June 2020).

